# Development of fistula between esophagogastric anastomotic site and cartilage portion of trachea after subtotal esophagectomy for cervical esophageal cancer: a case report

**DOI:** 10.1186/s40792-016-0238-2

**Published:** 2016-10-06

**Authors:** Daisuke Taniguchi, Hiroshi Saeki, Yuichiro Nakashima, Ryosuke Tsutsumi, Sho Nishimura, Kensuke Kudou, Yu Nakaji, Hirotada Tajiri, Satoshi Tsutsumi, Takafumi Yukaya, Ryota Nakanishi, Masahiko Sugiyama, Hideto Sonoda, Kippei Ohgaki, Eiji Oki, Yoshihiko Maehara

**Affiliations:** Department of Surgery and Science, Graduate School of Medical Sciences, Kyushu University, 3-1-1 Maidashi, Higashi-ku, Fukuoka 812-8582 Japan

**Keywords:** Fistula, Trachea, Anastomotic leak, Esophagectomy

## Abstract

A 65-year-old man with cT3N2M0 stage III cervical esophageal cancer underwent subtotal esophagectomy and gastric tube reconstruction through the retrosternal route after neoadjuvant chemoradiotherapy. The anastomosis was located adjacent to the left side of the trachea, and a circular stapler was used for anastomosis. Postoperative anastomotic leakage occurred, and an esophagotracheal fistula between the esophagogastric anastomotic site and cartilage portion of the trachea was observed on postoperative day 44. The patient underwent division of the fistula, direct suturing of the anastomotic leakage site, left pectoralis major muscle flap placement, and tracheotomy. He was discharged home on postoperative day 120 on an oral diet. All previous reports of tracheobronchial fistula describe the occurrence of the fistula at the membranous portion of the trachea. The formation of a fistula between the esophagogastric anastomotic site and cartilage portion of the trachea is considered a possible complication when a high esophagogastric anastomosis is created.

## Background

Tracheobronchial fistula is a rare but serious complication after esophageal surgery. It often results in respiratory failure, pulmonary sepsis, and finally septic shock and death [1]. Tracheobronchial fistula may occur secondary to anastomotic leakage with inflammatory involvement, ischemia of the tracheobronchial tree, extensive mediastinal node dissection, direct surgical injury to the tracheobronchial tree [1], tracheobronchial erosion caused by gastric staples [2], endoscopic dilatation of an anastomotic stricture [3], cuff-induced tracheal necrosis during prolonged endotracheal intubation, or fragility of the blood supply of the interposed conduit [4]. The optimal methods for early diagnosis and treatment of tracheobronchial fistula are controversial, and the literature on tracheobronchial fistula mainly comprises case reports because of the rarity of this life-threatening complication [4]. All previous reports of tracheobronchial fistula describe occurrence of the fistula at the membranous portion of the trachea-bronchus [4–9]. To the best of our knowledge, this is the first report of a patient who developed a fistula between the esophagogastric anastomotic site and the cartilage portion of the trachea after subtotal esophagectomy for cervical esophageal cancer.

## Case presentation

A 65-year-old man presented with a 2-month history of dysphagia. Endoscopy showed a neoplastic lesion at the esophageal inlet. Pathological examination with biopsy revealed moderately differentiated squamous cell carcinoma. Computed tomography (CT) showed esophageal wall thickening and superior mediastinal lymph node enlargement. CT also showed that the tumor was in close proximity to the thoracic vertebrae, but there was no evidence of invasion on magnetic resonance imaging. Positron emission CT showed no distant metastasis. The preoperative diagnosis was clinical T3N2M0 stage III cervical esophageal cancer according to the tumor, node, and metastasis classification [10]. The patient underwent preoperative chemotherapy (5-fluorouracil and cisplatin) and radiation therapy (41.4 Gy). Two months later, endoscopic examination showed that the neoplastic lesion had changed to scar tissue. CT showed a reduction in the esophageal wall thickness and size of the lymph nodes. The patient underwent subtotal esophagectomy, gastric tube reconstruction, and three-field lymph node dissection. The following lymph nodes in the neck and upper and middle mediastinum were dissected: the cervical paraesophageal (#101), supraclavicular (#104), upper thoracic paraesophageal (#105), recurrent nerve (#106recL and #106recR), tracheobronchial (#106tbL), subcarinal (#107), middle thoracic paraesophageal (#108), and main bronchus (#109 L and #109R) lymph nodes [10]. The right bronchial artery was ligated and cut in our usual manner. Severe fibrous change secondary to the neoadjuvant chemoradiotherapy was observed in the upper mediastinum. The gastric tube was pulled up to the neck incision through the retrosternal route. An anastomosis between the esophagus and gastric tube was created with a 21-mm-diameter circular stapler in an end-to-side fashion. The anastomotic position was located adjacent to the left side of the trachea. The intubation, anesthetic course, and surgical procedure were uneventful. Pathological examination of a resected specimen of the esophageal wall showed mild chronic inflammation, accompanied by dense transmural fibrosis and no carcinoma cells. All 71 lymph nodes were free of carcinoma cells. Curative resection (R0) was achieved.

**Fig. 1:**
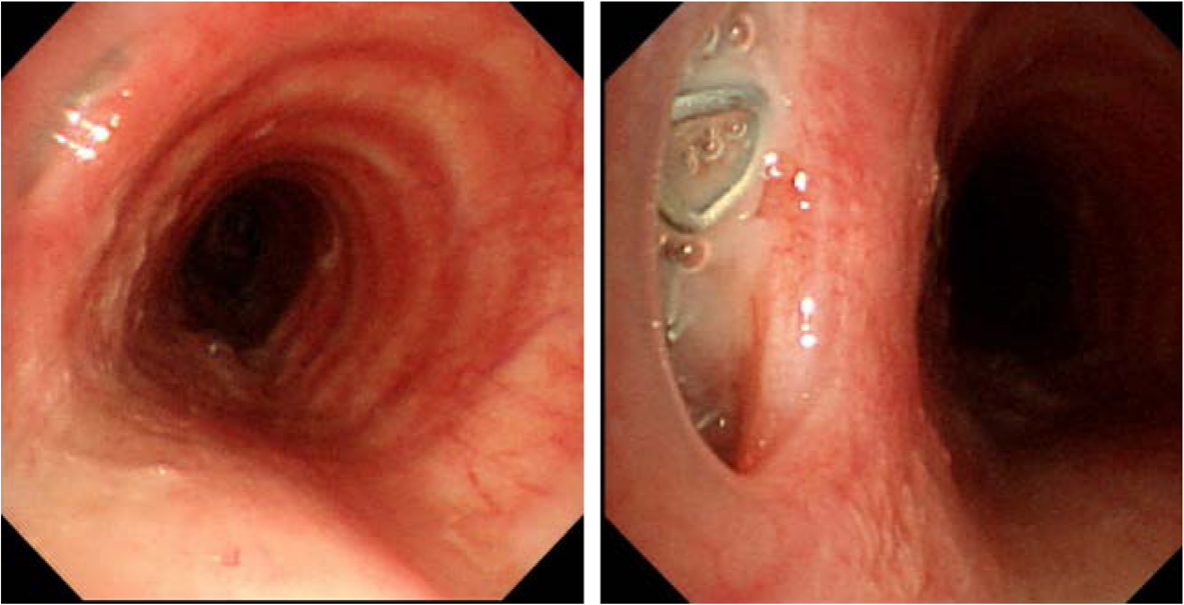
Bronchoscopy on postoperative day 44 revealed that the staples, used for the anastomosis, were exposed at the cartilage portion of the trachea

**Fig. 2:**
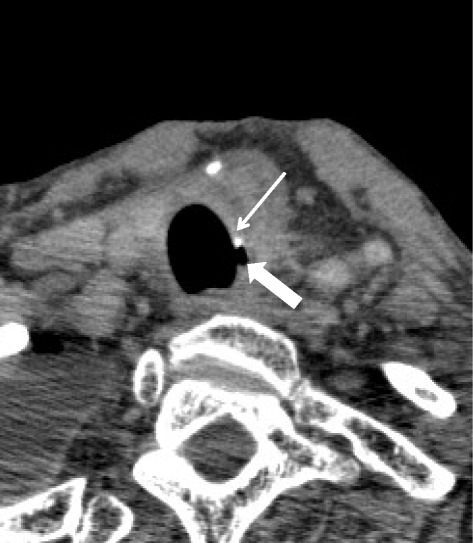
CT scan showed that a fistula (thick arrow) had developed on the left side cartilage portion of the trachea. A circular stapler (*thin arrow*) was also found at the fistula portion

**Fig. 3:**
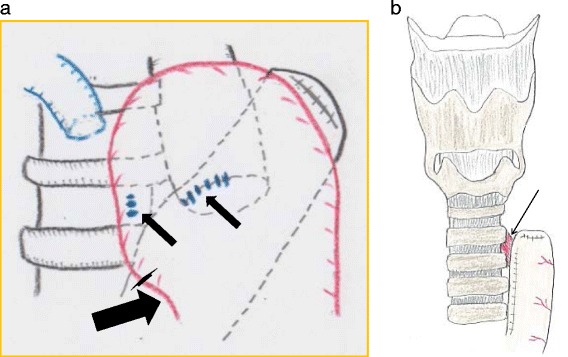
**a** Schema of the intraoperative findings. The anastomotic site fistula and the cartilage portion of the trachea were sutured (*thin arrows*) and a left pectoralis major muscle flap (*thin arrow*) covered the fistula. **b** Schema of the fistula between the high cervical anastomosis and the left-side tracheal cartilage

On postoperative day (POD) 5, purulent discharge was observed in the drainage tube inserted near the anastomotic site. CT revealed free gas adjacent to the left side of the cervical anastomotic site. Anastomotic leakage was suspected, and the patient was managed conservatively with antibiotics and, enteral nutrition via a jejunostomy. In spite of conservative therapy, the patient’s fever was prolonged and his respiratory condition gradually worsened. Bronchoscopy on POD 44 revealed that the staples originating from the circular stapler used for the anastomotic site or the linear stapler used for the lesser curvature were exposed at the cartilage portion of the trachea about 5 cm peripheral to the vocal cords (Fig. 1). CT showed a fistula at the left-side cartilage portion of the trachea (Fig. 2). The patient was treated under medical respiratory management and sent to the intensive care unit. Surgical division of the fistula without combined resection of the cartilage, direct suturing of the anastomotic leakage site, left pectoralis major muscle flap placement, and tracheotomy was performed on POD 48 (Fig. 3a). Surgical repair of the cartilage site was performed with whole layer interrupted 4-0 synthetic absorbent monofilament sutures crossing over two adjacent cartilages. The pectoralis major muscle flap was mobilized to the cervical site. The muscle flap covered the repair portion and was fixed to the surrounding tissue using 4-0 synthetic absorbent monofilament sutures. After surgical repair, blood tests showed improvement in the signs of inflammation, and the patient’s respiratory condition clinically improved. He was withdrawn from the respirator and returned to the general ward on POD 55. A gastrografin swallow study performed on POD 75 revealed no signs of anastomotic leakage or stenosis. He was introduced to an oral diet on POD 81. The patient was discharged home on POD 120 on an oral diet.

## Discussion

We have herein described a patient who developed a fistula between the esophagogastric anastomosis and cartilage portion of the trachea after esophagectomy for cervical esophageal cancer. Fistulae between the esophagogastric anastomotic site and trachea or bronchus after esophagectomy have been reported, but all occurred at the membranous portion of the tracheobronchus. The anastomotic site is often closed to the trachea anatomically, and an esophagotracheal fistula could occur at the fragile membranous portion of the trachea. In the current case, the gastric tube was pulled up through the retrosternal route, and the high cervical anastomosis was adjacent to the left side tracheal cartilage, not to the membranous portion (Fig. 3b). The anastomotic site was obviously higher than the usual anastomosis for thoracic esophageal cancer. We believe that this is the main reason why the esophagotracheal fistula formed at the cartilage portion, not at the membranous portion. This point also represents a significant anatomical difference between the current case and previously reported case involving a fistula at the membranous portion [4–9]. When we perform gastric tube reconstruction through the posterior mediastinal route and anastomosis with a linear stapler, the staple line on the lesser curvature of the gastric tube and anastomotic site can contact the membranous portion of the trachea. Because the membranous portion of the trachea is fragile, we routinely prevent the staple line from contacting the trachea. To accomplish this, the staple line on the gastric tube and anastomotic site is covered with seromuscular sutures and the greater omentum is interposed between the staple line and membranous portion of the trachea. However, when reconstruction is performed through the retrosternal route, the staple line usually does not contact the membranous portion of the trachea. In this case, we only covered the staple line located on the lesser curvature of the gastric tube with seromuscular sutures. The anastomotic site was not covered with seromuscular sutures, and the greater omentum was not interposed because we used a circular stapler; theoretically, the staples were not exposed outside of the anastomotic site.

According to some reports, the incidence of anastomotic leakage after esophagectomy ranges from 10 to 35 % [11, 12]. The degree of intraoperative gastric ischemia due to gastric tube creation is associated with the development of anastomotic complications [13]. Anastomotic leaks are responsible for approximately 40 % of post-esophagectomy deaths [11]. In the current case, we suspected that neoadjuvant chemoradiotherapy and high cervical anastomosis may have been the factors that caused the anastomotic leakage [14–16]. Neoadjuvant chemoradiotherapy is an independent risk factor for anastomotic leakage, as reported in previous literature [14–16]. Neoadjuvant chemoradiotherapy might affect the blood supply, immune system, or fibrosis of tissues, which might result in anastomotic leakage [15]. Peritracheal inflammation due to anastomotic leakage may result in a fistula to the trachea, which is probably the most important course of this complication [1]. Clinically, apparent thoracic anastomotic leaks and fistulae are associated with a high rate of mortality in spite of advances in critical care [17]. In this case, we considered the following possible mechanisms of development of the esophagotracheal fistula. First, inflammation caused by anastomotic leakage spread to the trachea. Second, the staples originating from the circular stapler used for the anastomotic site or from the linear stapler used for the lesser curvature became exposed following the anastomotic leakage and directly injured the trachea. In addition, the trachea was fragile or might have been injured because preoperative chemoradiotherapy might make intraoperative adhesiotomy difficult. When the anastomosis is adjacent to the trachea, even the cartilage portion of the trachea, vital tissue may need to be interposed between the trachea and anastomosis.

Although this is a serious complication, there is no standard management of esophagotracheal fistula. Therapy should depend on the site, size, underlying cause, and severity of symptoms. A conservative treatment regimen may be considered for patients with only mild symptoms. When conservative treatment fails or symptoms are more severe, surgical intervention is necessary. According to the literature, if the fistula fails to heal within a 4- to 6-week period, conservative treatment should be abandoned. Our patient developed a persistent cough and fever. Blood tests revealed prolonged inflammation. The patient’s respiratory condition gradually worsened, and he was considered to have pneumonia, pulmonary sepsis, and respiratory failure. We decided to perform an operation. An omental or pleural patch or a muscle flap can be applied to fill the dead space and add vital tissue to the defect, preventing recurrent fistulization [18, 19]. Because blood flow is poor in the area of the leakage, these vital soft tissues play an important role in wound healing and control of local infection. In our institute, a pectoralis major muscle flap has been used to repair prolonged anastomotic leakage after esophageal reconstruction, and we have been able to manage this serious complication successfully up to the present time [20]. The advantages of the pectoralis major muscle flap for head and neck reconstruction are well known; it is a readily available source of vascularized tissue and is easily harvested for use in the head and neck.

## Conclusions

We have herein described a patient who developed a fistula between the esophagogastric anastomotic site and cartilage portion of the trachea after esophagectomy for cervical esophageal cancer. This potentially life-threatening complication was successfully treated with surgical repair using a pectoralis major muscle flap. Basic management is the same even if the fistula site is at the membranous portion or cartilage portion of the trachea. However, we believe that this report is clinically valuable because the information provided by this report might help surgeons to make an early diagnosis and consequently provide appropriate treatment for this rare postoperative complication. When the anastomotic position is adjacent to the trachea, even the cartilage portion, an esophagotracheal fistula is a possible complication associated with anastomotic leakage.

## Abbreviations

CT: Computed tomography
POD: Postoperative day

## References

[1] Bartels HE, Stein HJ, Siewert JR (1998). Tracheobronchial lesions following oesophagectomy: prevalence, predisposing factors and outcome. Br J Surg..

[2] Pramesh CS, Sharma S, Saklani AP (2001). Broncho-gastric fistula complicating transthoracic esophagectomy. Dis Esophagus..

[3] Aguilo Espases R, Lozano R, Navarro AC (2004). Gastrobronchial fistula and anastomotic esophagogastric stenosis after esophagectomy for esophageal carcinoma. J Thorac Cardiovasc Surg..

[4] Schweigert M, Dubecz A, Beron M (2012). Management of anastomotic leakage-induced tracheobronchial fistula following oesophagectomy: the role of endoscopic stent insertion. Eur J Cardiothorac Surg..

[5] Marulli G, Loizzi M, Cardillo G (2013). Early and late outcome after surgical treatment of acquired non-malignant tracheo-oesophageal fistulae. Eur J Cardiothorac Surg..

[6] Maruyama K, Motoyama S, Okuyama M (2007). Esophagotracheal fistula caused by gastroesophageal reflux 9 years after esophagectomy. World J Gastroenterol..

[7] Hayashi K, Ando N, Ozawa S (1999). Gastric tube-to-tracheal fistula closed with a latissimus dorsi myocutaneous flap. Ann Thorac Surg..

[8] Song SW, Lee HS, Kim MS (2006). Repair of gastrotracheal fistula with a pedicled pericardial flap after Ivor Lewis esophagogastrectomy for esophageal cancer. J Thorac Cardiovasc Surg..

[9] Marty-Ané CH, Prudhome M, Fabre JM (1995). Tracheoesophagogastric anastomosis fistula: a rare complication of esophagectomy. Ann Thorac Surg..

[10] Japan Esophageal Society. Japanese classification of esophageal cancer. 10^th^ ed. Tokyo: Kanehara; 2008.

[11] Alanezi K, Urschel JD (2004). Mortality secondary to esophageal anastomotic leak. Ann Thorac Cardiovasc Surg..

[12] Blencowe NS, Strong S, McNair AG (2012). Reporting of short-term clinical outcomes after esophagectomy: a systematic review. Ann Surg..

[13] Pham TH, Perry KA, Enestvedt CK (2011). Decreased conduit perfusion measured by spectroscopy is associated with anastomotic complications. Ann Thorac Surg..

[14] Saeki H, Morita M, Tsuda Y (2013). Multimodal treatment strategy for clinical T3 thoracic esophageal cancer. Ann Surg Oncol..

[15] Tachimori Y, Kanamori N, Uemura N (2009). Salvage esophagectomy after high-dose chemoradiotherapy for esophageal squamous cell carcinoma. J Thorac Cardiovasc Surg..

[16] Morita M, Masuda T, Okada S (2009). Preoperative chemoradiotherapy for esophageal cancer: factors associated with clinical response and postoperative complications. Anticancer Res..

[17] Junemann-Ramirez M, Awan MY, Khan ZM (2005). Anastomotic leakage post-esophagogastrectomy for esophageal carcinoma: retrospective analysis of predictive factors, management and influence on longterm survival in a high volume centre. Eur J Cardiothorac Surg..

[18] Morita M, Saeki H, Okamoto T (2015). Tracheobronchial fistula during the perioperative period of esophagectomy for esophageal cancer. World J Surg..

[19] Buskens CJ, Hulscher JB, Fockens P (2001). Benign tracheoneo-esophageal fistulas after subtotal esophagectomy. Ann Thorac Surg..

[20] Morita M, Ikeda K, Sugiyama M, Saeki H (2010). Repair using the pectoralis major muscle flap for anastomotic leakage after esophageal reconstruction via the subcutaneous route. Surgery..

